# HANNA: hard-constraint neural network for consistent activity coefficient prediction†

**DOI:** 10.1039/d4sc05115g

**Published:** 2024-10-31

**Authors:** Thomas Specht, Mayank Nagda, Sophie Fellenz, Stephan Mandt, Hans Hasse, Fabian Jirasek

**Affiliations:** a Laboratory of Engineering Thermodynamics (LTD), RPTU Kaiserslautern Germany fabian.jirasek@rptu.de; b Department of Computer Science, RPTU Kaiserslautern Germany; c Department of Computer Science, University of California Irvine CA USA

## Abstract

We present the first hard-constraint neural network model for predicting activity coefficients (HANNA), a thermodynamic mixture property that is the basis for many applications in science and engineering. Unlike traditional neural networks, which ignore physical laws and result in inconsistent predictions, our model is designed to strictly adhere to all thermodynamic consistency criteria. By leveraging deep-set neural networks, HANNA maintains symmetry under the permutation of the components. Furthermore, by hard-coding physical constraints in the model architecture, we ensure consistency with the Gibbs–Duhem equation and in modeling the pure components. The model was trained and evaluated on 317 421 data points for activity coefficients in binary mixtures from the Dortmund Data Bank, achieving significantly higher prediction accuracies than the current state-of-the-art model UNIFAC. Moreover, HANNA only requires the SMILES of the components as input, making it applicable to any binary mixture of interest. HANNA is fully open-source and available for free use.

## Abstract 2

We introduce HANNA, the first hybrid neural network model that strictly complies with all thermodynamic consistency criteria for predicting activity coefficients and outperforms current benchmark methods in terms of accuracy and applicability.

## Graphical abstract

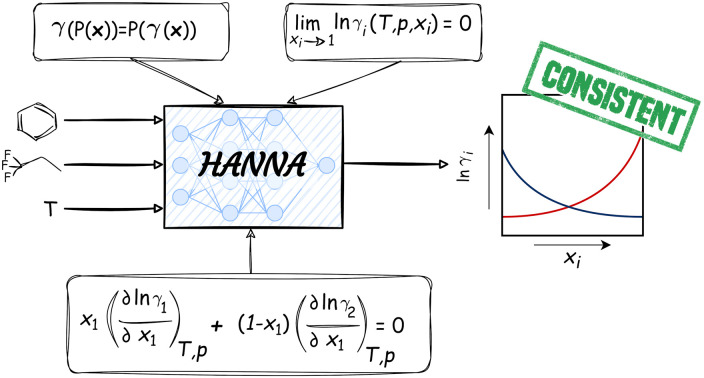

## Introduction

Neural networks (NNs) have recently revolutionized many fields, including image analysis,^1^ speech recognition,^2^ predicting protein folding,^3,4^ and language modeling.^5,6^ These models are universal and highly flexible function approximators,^7^ which perform best if they have large amounts of training data. NNs are also gaining more and more attention in chemical engineering^8–12^ but face two significant challenges preventing them from exploiting their full potential in this field: sparse training data and inconsistent predictions. Like in other fields of science and engineering, data sparsity is ubiquitous in chemical engineering due to the high effort and costs related to experimental data collection, making predictions with purely data-driven NNs difficult. Furthermore, since NNs are *a priori* agnostic about physical laws and boundaries, there is no guarantee that their predictions obey these rules, frequently leading to physically inconsistent results and predictions.^13^ This, in turn, is detrimental to the trust in NN-based models and a severe obstacle to their adoption and use in practice.

The most promising solution to these challenges is to incorporate explicit physical knowledge into NNs to support their training beyond using only the limited available data. Most prominently, Physics-Informed Neural Networks (PINNs)^14^ have been successfully applied in different fields,^10,13,15–20^ primarily to solve partial differential equations (PDE) efficiently. PINNs incorporate the governing physical equation or boundary conditions into the loss function of an NN by adding a term that penalizes solutions deviating from the constraint (*e.g.*, the compliance of a PDE).^21^ PINNs are inherently soft-constraint methods that do not enforce *exact* compliance with the given constraints, which is a well-known limitation of penalty methods in general^22,23^ and has potential drawbacks. Specifically, while approximately complying with physical laws and boundaries might be sufficient in some cases, this is unacceptable in many applications; for instance, thermodynamic models that yield physically inconsistent predictions will not be accepted and used in chemical engineering practice.

Hard-constraint models, which strictly enforce physical relations and constraints in NNs, are generally considered challenging to develop.^21,23–26^ Thermodynamics is the ideal field for designing such hard-constraint models with its extensive treasure of explicit physical knowledge on the one hand and the high demand for strict compliance of predictive thermodynamic models with physical laws and constraints on the other. In this work, we introduce the first hard-constraint NN-based model for thermodynamic property prediction, which opens up an entirely new way of thermodynamic model development but also holds the promise to advance model development in other fields of chemical engineering and beyond.

Predicting the thermodynamic properties of pure components and mixtures is fundamental in many fields of science and engineering. In chemical engineering, knowledge of thermodynamic properties is the basis for process design and optimization. However, experimental data on thermodynamic properties are scarce. The problem is particularly challenging for mixtures, where missing data are prevalent due to the combinatorial complexity involved.

One of the most critical thermodynamic properties is the activity coefficient of a component in a mixture. Activity coefficients are the key to modeling the chemical potential in liquid mixtures, one of the most central properties in physical chemistry and chemical engineering. Activity coefficients are essential for correctly describing chemical equilibria,^27^ reaction kinetics,^28^ phase equilibria,^29^ and many other properties of mixtures, such as electrochemical properties.^30,31^ Since activity coefficients cannot be measured directly, they are usually determined indirectly by evaluating phase equilibrium experiments. Since these experiments are time-consuming and expensive, experimental data on activity coefficients are often lacking, and many physical prediction methods have been developed and are widely applied in industry.^29^

Physical methods for predicting activity coefficients model the molar Gibbs excess energy *g*^E^ as a function of temperature *T* and mixture composition in mole fractions ***x***, from which the logarithmic activity coefficients ln *γ*_*i*_ are obtained by partial differentiation.^29^ The two most widely used *g*^E^ models are NRTL^32^ and UNIQUAC.^33^ These models generalize over state points, *i.e.*, temperature and mole fractions, but cannot extrapolate to unstudied mixtures. In contrast, *g*^E^ models based on quantum-chemical descriptors, such as COSMO-RS^34^ and COSMO-SAC,^35–37^ or group-contribution models, such as the different versions of UNIFAC^38,39^ (with modified UNIFAC (Dortmund) being the most advanced^39,40^) also allow to generalize over components and mixtures. However, even though they have been continuously developed and refined for decades, the state-of-the-art models show significant weaknesses for certain classes of components. The consequential inaccuracies in predicting activity coefficients result in wrongly predicted phase equilibria, leading to poor process modeling and simulation.^41,42^ On the upside, the theoretical foundation of the established physical models allows for good extrapolation performance, and, even more importantly, they exhibit strict compliance with thermodynamic laws, boundaries, and consistency criteria.

Recently, machine-learning (ML) methods have gained attention for predicting activity coefficients^43–45^ and other thermodynamic properties.^46–52^ Even though these models are purely data-driven, they surpassed the physical thermodynamic models in prediction accuracy. However, they were all limited to specific state points and could, *e.g.*, not describe the composition dependence of activity coefficients.

To improve the ML models further, various hybridization approaches^53^ were developed that combine the flexibility of ML methods with physical knowledge. This was, *e.g.*, done by augmenting the training data with synthetic data obtained from physical prediction methods.^42,54^ Other recently developed hybridization approaches^55–57^ have broadened the application range of physical thermodynamic models. In these approaches, an ML method is embedded in a physical thermodynamic model to predict the physical model's parameters. By retaining the framework of the physical models, these hybrid models are intrinsically thermodynamically consistent. On the downside, these models are subject to the same assumptions and simplifications taken during the development of the original model, limiting their flexibility. Consequently, they have a restricted value range of predictable activity coefficients,^58^ limiting the description of certain phase behaviours.^59–62^

Rittig *et al.* recently developed a PINN^13^ and a hard-constraint approach^63^ considering the Gibbs–Duhem equation; however, their study was limited to synthetic data and the Gibbs–Duhem equation as only one of the relevant physical boundary conditions. Hybrid models for activity coefficient prediction that *fully* exploit the flexibility of NNs while *guaranteeing* consistency with *all* thermodynamic constraints have not been available until now. This work has addressed this gap.

Specifically, we have developed the first hard-constraint NN model for the Gibbs excess energy *g*^E^ of a mixture, which allows us to predict activity coefficients ln *γ*_*i*_ in any binary mixture of arbitrary components at any state point. We name our method **HA**rd-constraint **N**eural **N**etwork for **A**ctivity coefficient prediction (HANNA) in the following. We restrict ourselves here to binary mixtures. All physical models of mixtures are based on pair interactions, which can, and practically always are, trained on data for binary mixtures. Therefore, predictions for binary activity coefficients obtained from HANNA could be used to fit the parameters of a physical model based on pair-interactions, which can then be used for predictions of multicomponent mixtures. However, it would also be very interesting to study the generalization of HANNA to multicomponent mixtures in future work.

## Development of HANNA

HANNA combines a flexible neural network with explicit physical knowledge. At its heart, it predicts the Gibbs excess energy *g*^E^ of a mixture, from which subsequently the activity coefficients of the mixture components, typically given in the natural logarithm ln *γ*_*i*_, can be derived. The Gibbs excess energy *g*^E^ and consequently the activity coefficients ln *γ*_*i*_, are typically expressed as functions of temperature *T*, pressure *p*, and the composition in mole fractions ***x*** of the components. In the following, we will express *g*^E^ and the activity coefficients ln *γ*_*i*_ in binary mixtures as functions of *T*, *p*, and *x*_1_. For liquid mixtures, the influence of the pressure is small and is often neglected, which is also the case for our model. However, for the sake of clarity, all thermodynamic derivations are written here without this assumption.

The predictions of HANNA strictly comply with all relevant thermodynamic consistency criteria, which are listed for binary mixtures as follows.

(1) The activity coefficients of pure components are unity:1
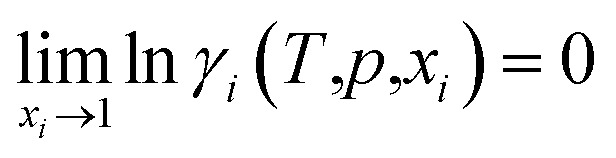


(2) The activity coefficients of the components in a mixture are coupled by the Gibbs–Duhem equation, which reads for the binary mixture:2
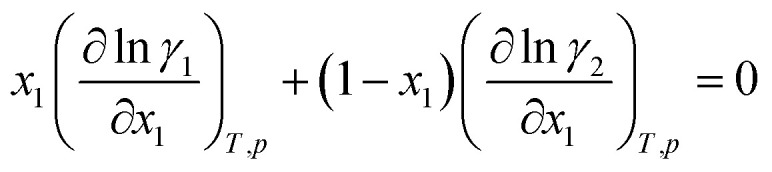


(3) The activity coefficients in a pseudo-binary mixture *A* + *B* where *A* = *B* are always unity:

3 ln *γ*_*i*_(*T*,*p*,*x*_*i*_) = 0

(4) Upon changing the order of the components in the input of a model for predicting the activity coefficients ln *γ*_1_ and ln *γ*_2_ in a binary mixture, the values of the predicted activity coefficients must not change, only their order. Mathematically, this is called permutation-equivariance and can be expressed as:

4 ***γ***(*P*(***x***)) = *P*(***γ***(***x***))

where ***γ*** is the vector containing the (logarithmic) activity coefficients of the mixture components, ***x*** is the vector containing the information on the components in the input, including their descriptors and mole fractions, and *P* is a permutation operator.

**Fig. 1:**
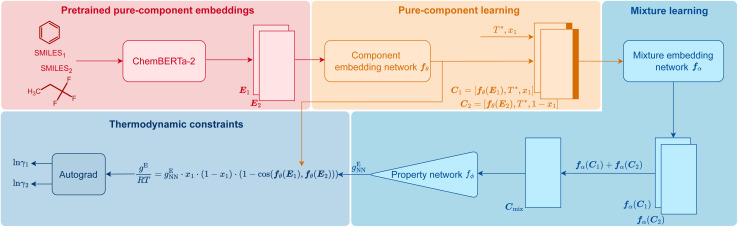
Scheme of HANNA, the first hard-constraint NN for predicting activity coefficients in binary mixtures. Technical details on the architecture are given in Section *Data splitting, training, and evaluation of the model*.

In Fig. 1, we visualize how HANNA strictly enforces these constraints for predicting activity coefficients, leading to the novel class of hybrid NNs developed in this work. The central idea is to learn the molar excess Gibbs energy *g*^E^ of the mixture rather than the individual activity coefficients (*γ*_1_ and *γ*_2_) directly. The values of *γ*_1_ and *γ*_2_ can then be obtained from *g*^E^ by the laws of thermodynamics, ensuring strict thermodynamic consistency. HANNA consists of four parts:

### (1) Pure-component embeddings from pretrained ChemBERTa-2

We use SMILES^64^ strings to represent the components and preprocess them with ChemBERTa-2,^65^ a language model pretrained on an extensive database of molecules for learning “pure component embeddings” of the molecules from the respective SMILES.

### (2) Refining pure-component embeddings for thermodynamic property prediction

Since the embeddings of ChemBERTa-2 were not explicitly trained on thermodynamic properties, we “fine-tune” them to predict thermodynamic properties in a two-step process. We first feed them into a “component embedding network” ***f***_*θ*_ to get a lower dimensional representation of each component *i*. Then, the information on the standardized temperature T* (see Section *Data splitting, training, and evaluation of the model* for the definition) and the composition (here: mole fraction *x*_1_ of component 1) are concatenated to each of the component embeddings. The result of this step is a refined embedding for each component *i*, represented as vector ***C***_*i*_, tailored for thermodynamic mixture property prediction.

### (3) Learning mixture embeddings and preliminarly prediction

The component embeddings ***C***_*i*_ are then individually processed by the “mixture embedding network” ***f***_*α*_, whose outputs are then aggregated using the sum operation to yield ***C***_mix_. This step guarantees permutation invariance, *i.e.*, independence of the order of the components, an idea inspired by deep-set models,^66,67^ and ensures that eqn (4) is fulfilled. Subsequently, the sum is fed into another “property prediction” network *f*_*ϕ*_ whose output *g*^E^_NN_ is a scalar that can be understood as a preliminary prediction of the molar Gibbs excess energy *g*^E^ of the mixture.

### (4) Enforcing all physical consistency criteria

In this step, *g*^E^_NN_ is further processed to guarantee the compliance of HANNA's predictions with the remaining consistency criteria, *cf.*eqn (1)–(3). Step 4 basically corrects the preliminary *g*^E^_NN_ to hard-constrain the final predicted molar Gibbs excess energy *g*^E^ on physically consistent solutions. Specifically, *g*^E^ of the mixture of interest is calculated by:5
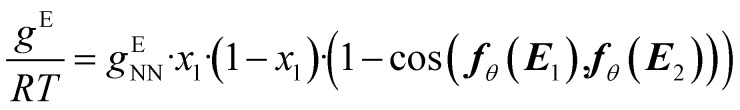
where6
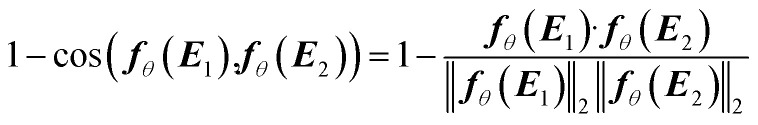
denotes the cosine distance between the two component embeddings ***f***_*θ*_(***E***_1_) and ***f***_*θ*_(***E***_2_), *R* is the ideal gas constant, and *T* is the absolute temperature in Kelvin. The term *x*_1_·(1 − *x*_1_) in eqn (5) ensures that *g*^E^ becomes zero in the case of pure components (*x*_1_ = 1 or *x*_1_ = 0), thereby enforcing strict consistency with regard to eqn (1). The cosine distance, *cf.*eqn (6), ensures that if the two component embeddings are identical, *i.e.*, the studied “mixture” is, in fact, a pure component (cosine distance equals zero), *g*^E^ always becomes zero to guarantee consistency regarding eqn (3).

Finally, the logarithmic activity coefficients ln *γ*_*i*_ are derived in a thermodynamically consistent way from *g*^E^ by partial differentiation, which reads for a binary mixture:^29,68^7
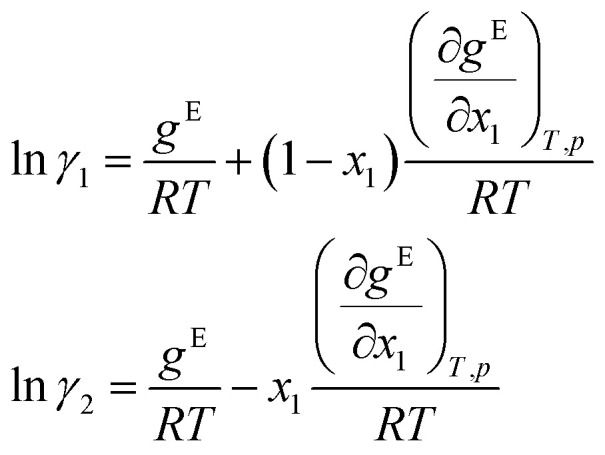


For this purpose, the auto-differentiation function “autograd” from pytorch^69^ is used to calculate ln *γ*_*i*_ following eqn (7). This last step intrinsically ensures the Gibbs–Duhem consistency of the predicted activity coefficients, *cf.*eqn (2). Furthermore, since *g*^E^ is enforced to be permutation-invariant in step 3, the differentiation in eqn (7) always yields permutation-equivariant predictions for ln *γ*_*i*_.

HANNA was trained end-to-end and evaluated on 317 421 data points for ln *γ*_*i*_ in 35 012 binary systems from the Dortmund Data Bank (DDB),^70^*cf.* Section *Data* for details. The data set was randomly split system-wise in 80% training, 10% validation, and 10% test set. Technical details on HANNA and the optimization procedure are given in Section *Data splitting, training, and evaluation of the model*. We also trained and validated a version of HANNA on 100% of the data with the final set of hyperparameters. This version is not discussed or used to evaluate the predictive performance of HANNA in this work but will be provided together with this paper as an open-source version. This final version of HANNA should be used if activity coefficients in any binary mixture need to be predicted. The only inputs needed are the SMILES of the components, their mole fractions, and the temperature.

## Results

In the following, we discuss the performance of HANNA for predicting activity coefficients from the test set, which were not used for training or hyperparameter optimization. For comparison, we also include the results of modified UNIFAC (Dortmund),^39,40^ referred to simply as UNIFAC in the following. The UNIFAC training set has not been disclosed. However, since the groups developing UNIFAC and maintaining the DDB are essentially the same, one can assume that a large share of the data considered here was also used for training UNIFAC. Hence, contrary to the results of HANNA, the results obtained with UNIFAC cannot be considered true predictions. This generates a strong bias of the comparison in favor of UNIFAC.

We compare the performance of the models using a system-wise error score. Specifically, we calculate system-specific mean absolute errors (MAE) by averaging the absolute deviations of the predicted logarithmic activity coefficients from the experimental data for each system from the test set. This procedure ensures equal weighting of all systems irrespective of the number of data points and prevents overweighting well-studied systems like water + ethanol. All 3502 systems in the test set can be predicted with HANNA, but due to missing parameters, only 1658 can be modeled with UNIFAC. Therefore, both models are compared on the smaller shared horizon, called the “UNIFAC horizon” in the following.

**Fig. 2:**
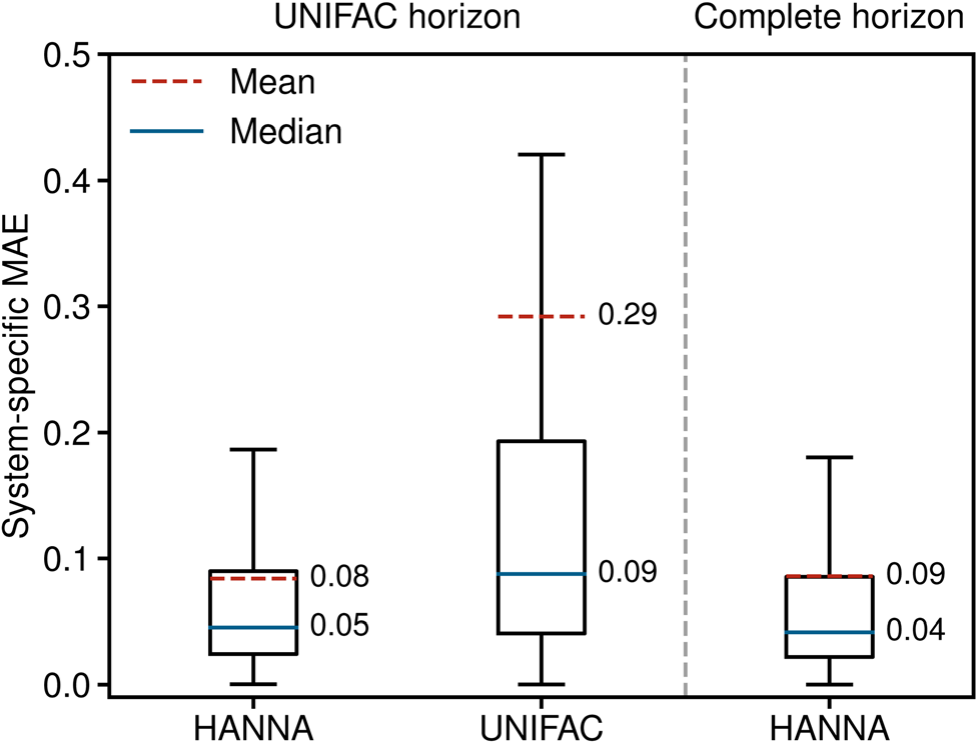
System-specific MAE of the predicted logarithmic activity coefficients ln *γ*_*i*_ from HANNA and UNIFAC. Left: results for those data from the test set that can also be predicted with UNIFAC (UNIFAC horizon). Right: results for the complete test set (complete horizon).

Fig. 2 shows the system-specific MAE of the predicted logarithmic activity coefficients in boxplots; the whisker length is 1.5 times the interquartile range. Outliers are not depicted for improved visibility. The left panel of Fig. 2 shows the results for the UNIFAC horizon, *i.e.*, for the data points that can be predicted with both models. HANNA significantly outperforms UNIFAC, with a mean MAE reduced to approximately a third of UNIFAC's, particularly indicating a reduced number of very poorly predicted data points. Furthermore, the significantly reduced median MAE (from 0.09 to 0.05) indicates higher overall accuracy than UNIFAC. Fig. 2 (right) shows that the performance of our model on all test data (“complete horizon”), including those that cannot be predicted with UNIFAC, is similar to the UNIFAC-horizon performance. In Fig. S.7 in the ESI,† we show the robustness of HANNA over different random seeds for data splitting.

As each data point in the test set corresponds to a binary system, three different cases can occur:

(1) Only the combination of the two components is new, *i.e.*, the respective system was not present in the training or validation data. However, for both components, some data (in other systems) were used for training or validation.

(2) One component is unknown, *i.e.*, only for one of the components, some data (in other systems) were used during training or validation.

(3) Both components are unknown, *i.e.*, no data for any of the components (in any system) were used during training or validation.

While we do not differentiate between these cases in Fig. 2, we demonstrate in Fig. S.6 in the ESI† that HANNA significantly outperforms UNIFAC in extrapolating to unknown components.

**Fig. 3:**
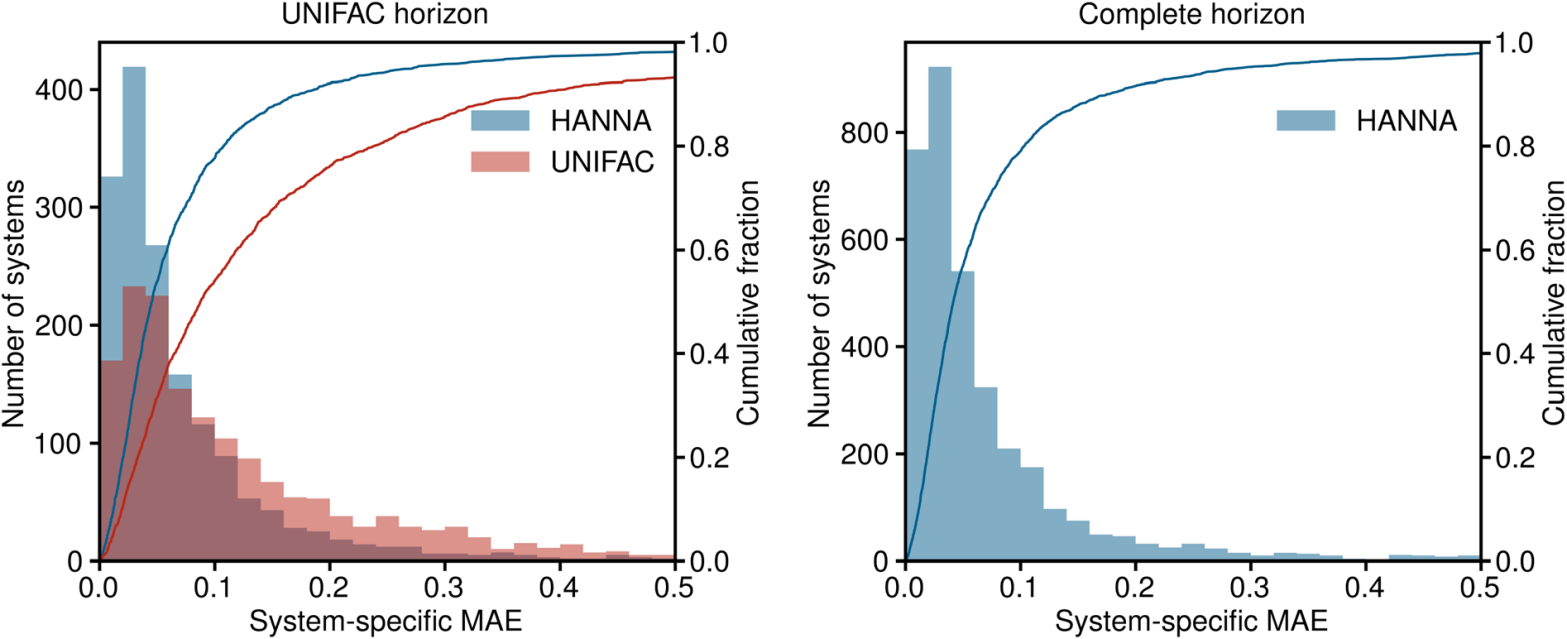
Histograms and cumulative fractions (lines) showing the system-specific MAE for predicting logarithmic activity coefficients ln *γ*_*i*_. Left: comparison of HANNA with UNIFAC on those test data that can be predicted with UNIFAC (UNIFAC horizon). The shown range covers 98.1% of the predictions of HANNA and 93.2% of the predictions of UNIFAC. Right: results of HANNA on the complete test set. The shown range covers 97.9% of the predictions.

In Fig. 3, the results for the test set are shown in a histogram representation of the system-specific MAE. Furthermore, the cumulative fraction, *i.e.*, the share of all test systems that can be predicted with an MAE smaller than the indicated value, is shown in Fig. 3. Again, in the left panel, the predictions of HANNA are compared to those of UNIFAC on the UNIFAC horizon; in the right panel, the predictions of HANNA for the complete test set are shown. The results underpin the improved prediction accuracy of HANNA compared to UNIFAC, *e.g.*, while approximately 78% of the test systems on the UNIFAC horizon can be predicted with an MAE < 0.1 with HANNA, which is in the range of typical experimental uncertainties for activity coefficients, this is the case for only approximately 54% with UNIFAC.

**Fig. 4:**
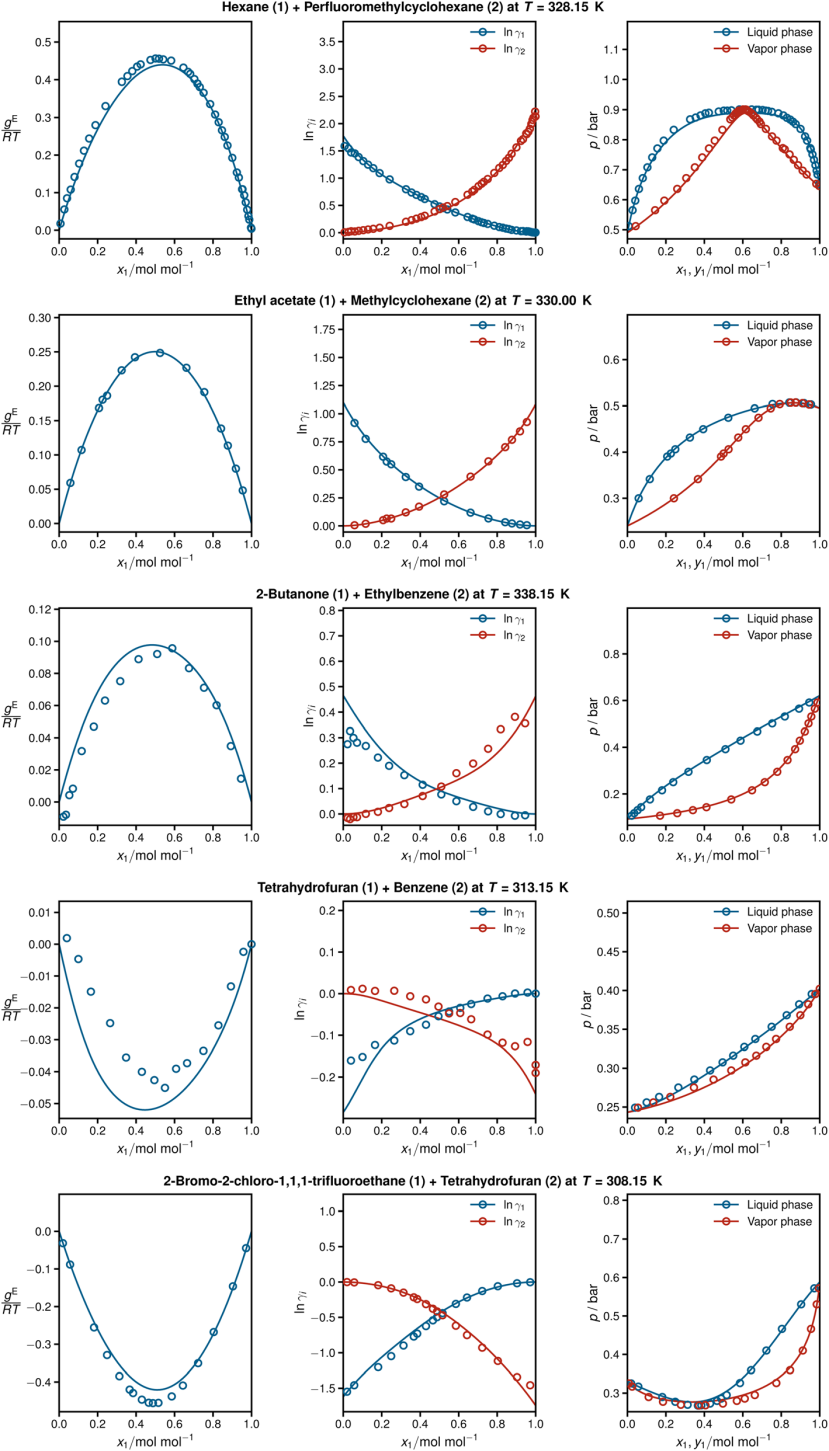
From left to right: Gibbs excess energies 
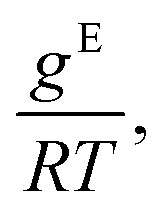
 resulting logarithmic activity coefficients ln *γ*_*i*_, and isothermal vapor–liquid phase diagrams for five systems from the test set plotted as a function of *x*_1_ as predicted with HANNA (lines) and comparison to experimental test data from the DDB^70^ (symbols). No data for any of the depicted systems were used for training or hyperparameter optimization.

Fig. 4 shows detailed results for five isothermal systems of the test set. In addition to the predicted activity coefficients as a function of the composition of the mixtures (middle panel), the corresponding Gibbs excess energies are plotted (left panel), which are internally predicted in HANNA, *cf.*Fig. 1. Furthermore, the respective vapor–liquid phase diagrams obtained with the predicted activity coefficients are shown (right panel), *cf.* Section *Data* for computational details. In all cases, HANNA's predictions (lines) are compared to experimental test data (symbols) from the DDB.

The shown systems were chosen randomly from the test set, aiming to cover various phase behaviours from low-boiling azeotropes (top), through approximately ideal systems (middle), to high-boiling azeotropes (bottom). In all cases, excellent agreement is found between the predictions and the experimental data. The results also demonstrate the thermodynamic consistency of HANNA: *g*^E^ = 0 and ln *γ*_*i*_ = 0 for the pure components, and the Gibbs–Duhem equation is fulfilled throughout.

In Section *Ablation studies* in the ESI,† results of ablation studies for which different parts in HANNA have been removed are presented. These results demonstrate the importance of hard-coding physical knowledge in the architecture of HANNA, not only regarding the thermodynamic consistency of the predictions but also regarding the predictive accuracy. Overall, the results clearly underpin the power of the hybrid approach, which combines the strengths of flexible NNs with that of physical knowledge. Given that our space of possible binary mixtures is easily in the millions, even if we only take components with experimental data on activity coefficients into account, it is remarkable that HANNA can generalize well based on only a fraction of about 1% of the binary systems.

## Conclusion

This work introduces a novel type of thermodynamic models: a hard-constraint neural network (NN) model combining the flexibility of NNs with rigorous thermodynamics. We demonstrate this for an essential thermodynamic modeling task: predicting activity coefficients in binary mixtures. The new hybrid model, HANNA, incorporates thermodynamic knowledge directly into the NN architecture to ensure strict thermodynamic consistency. HANNA was trained end-to-end on comprehensive data from the Dortmund Data Bank (DDB).

HANNA enables thermodynamically consistent predictions for activity coefficients in any binary mixture whose components can be represented as SMILES strings. It is fully disclosed and can be used freely. The predictive capacity of HANNA was demonstrated using test data from the DDB that were not used in model development and training. HANNA clearly outperforms the best physical model for predicting activity coefficients, modified UNIFAC (Dortmund), not only in terms of prediction accuracy but also regarding the range in which it can be applied, which is basically unlimited for HANNA but restricted for UNIFAC by the availability of parameters. Only about 50% of the mixtures in the test data set could be modeled with UNIFAC, while all could be predicted with HANNA.

Now that the path for developing hard-constraint NNs in thermodynamics is clear, many exciting options exist. As the framework presented here is based on the Gibbs excess energy, the Gibbs–Helmholtz equation is implicitly considered so that HANNA can be easily extended to also include excess enthalpies, which is expected to improve the description of the temperature dependence of the activity coefficients. Furthermore, not only enthalpies of mixing could be incorporated, but other types of thermodynamic data could also be used, *e.g.*, activity coefficients determined from liquid–liquid equilibria. The approach described here could also be extended to multicomponent mixtures. However, this can already be achieved by using HANNA to predict the binary subsystems and employing established physical models based on pair interactions for extrapolating to multicomponent mixtures.

Finally, the approach described here for Gibbs excess energy models can also be transferred to other thermodynamic modeling approaches, *e.g.*, equations of state based on the Helmholtz energy. More broadly, it could be adapted to merge physical theory with NNs in other scientific fields.

## Methods

### Data

Experimental data on vapor–liquid equilibria (VLE) and activity coefficients at infinite dilution in binary mixtures were taken from the Dortmund Data Bank (DDB).^70^ In preprocessing, data points labeled as poor quality by the DDB were excluded. Furthermore, only components for which a canonical SMILES string could be generated with RDKit^71^ from mol-files from DDB were considered.

From the VLE data, activity coefficients were calculated with extended Raoult's law:8
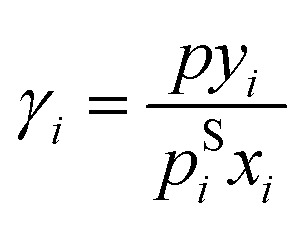
where *γ*_*i*_ is the activity coefficient of component *i* in the mixture, *x*_*i*_ and *y*_*i*_ are the mole fractions of component *i* in the liquid and vapor phase in equilibrium, respectively, *p* denotes the total pressure, and *p*^S^_*i*_ is the pure-component vapor pressure of *i*, which was computed using the Antoine equation with parameters from the DDB. The vapor phase was treated as a mixture of ideal gases in all cases. Furthermore, the pressure dependence of the chemical potential in the liquid phase was always neglected. Consequently, VLE data points at total pressures above 10 bar were excluded. The activity coefficients at infinite dilution, also normalized according to Raoult's law, were adopted from the DDB. The VLE diagrams in Fig. 4 were predicted using eqn (8) with the activity coefficients from HANNA and pure-component vapor pressures from the DDB.

The final data set after preprocessing comprises 317 421 data points and covers 35 012 binary systems and 2677 individual components.

### ChemBERTa-2 embeddings

The numerical embeddings of the components were generated from a pretrained language model called ChemBERTa-2,^65^ which was trained on a large database of SMILES. We used the “77M-MTR” model that is openly available on Huggingface.^72^ The “77M-MTR” model used 77 million SMILES to train ChemBERTa-2 in a multiregression task using the CLS token embedding.^65^ We use the CLS token embedding of the last layer of ChemBERTa-2, which results in a 384-dimensional input vector ***E***_*i*_ for each pure component *i*, *cf.*Fig. 1. The maximum number of tokens, *i.e.*, the individual SMILES building blocks used by ChemBERTa-2, was set to 512. The tokenization process of the original ChemBERTa-2 was slightly adapted here as explained in detail in Section *Improved tokenization of ChemBERTa-2* in the ESI† due to an error in the default tokenizer.

### Data splitting, training, and evaluation of the model

For training and evaluating the hybrid model HANNA, the data set was split randomly system-wise as follows: all data points for 80% of the binary systems (28 009) were used for training, all data points for another 10% of the systems (3501) were used for validation and hyperparameter optimization, and all data points for the remaining 10% of the systems (3502) were used to test the model. The data split was carried out system-wise, *i.e.*, all data points for an individual system are only present in a single set (training, validation, or test). This procedure ensures a fair evaluation of our model on truly unseen systems in the test set. The splitting of the systems to the different sets was completely random. In Fig. S.7 in the ESI,† we demonstrate the robustness of HANNA for different random splittings of the data set.

All models and training and evaluation scripts were implemented in Python 3.8.18 using PyTorch 2.1.2.^69^ The models were trained on one A40 GPU using the AdamW^73^ optimizer with an initial learning rate of 0.0005 or 0.001, a learning rate scheduler with a decay factor of 0.1, and a patience of 10 epochs based on the validation loss. The training was stopped if the validation loss (*cf.* below) was not improving for 30 epochs (early stopping), and the model with the best validation loss was chosen. Typical training times for the model were between 30 and 60 minutes.

The pure-component embedding network ***f***_*θ*_ and the property network *f*_*ϕ*_ consist of one hidden layer, whereas the mixture embedding network ***f***_*α*_ consists of two hidden layers, *cf.*Fig. 1. In all cases, the Sigmoid Linear Unit (SiLU) function with default PyTorch settings was used as the activation function.

The models are using the same number of nodes in each layer, except for the mixture embedding network ***f***_*α*_, where the input size is increased by two to include the standardized temperature and mole fraction of the respective component. Also, the output dimension of the property network *f*_*ϕ*_ is always one.

The embeddings of ChemBERTa-2 and the temperature in the training set were standardized using the StandardScaler from scikit-learn 1.3.0,^74^ whereas the mole fractions remained unchanged. The loss function SmoothL1Loss from PyTorch^69^ was used to mitigate the effect of experimental outliers of the activity coefficients. The hyperparameter *β* that controls the change between the L2 and L1 loss in the SmoothL1Loss was set to 0.25 and not varied. A batch size of 512 was used. The AdamW optimizer was used to update the NN weights during training. Besides the early stopping, the validation loss was used for hyperparameter tuning. The only varied hyperparameters were the weight decay parameter *λ* in the AdamW optimizer, the number of nodes in each network, and the initial learning rate, *cf.* above. Based on the results of the validation set, *λ* = 0.01 and 96 nodes with an initial learning rate of 0.001 were chosen. In the ESI† in Section *Hyperparameter optimization*, we discuss the influence of the different hyperparameters and present the validation loss results.

We provide a “final” version of HANNA with this paper that was trained as described above, except that no test set was used, *i.e.*, 90% of all systems were used for training and 10% for validation.

## Data availability

All data were taken from the Dortmund Data Bank.^70^ The final version of HANNA, which was trained and validated on 100% of the data (without using a test set), is available on Github (https://github.com/tspecht93/HANNA) and distributed under the MIT license.

## Author contributions

**Thomas Specht**: formal analysis, conceptualization, methodology, software, data curation, validation, writing – original draft, writing – review & editing, visualization. **Mayank Nagda**: methodology, software. **Sophie Fellenz**: formal analysis, writing – review & editing, methodology. **Stephan Mandt**: formal analysis, writing – review & editing, methodology, conceptualization. **Hans Hasse**: formal analysis, conceptualization, writing – review & editing, supervision, visualization, funding acquisition. **Fabian Jirasek**: formal analysis, conceptualization, methodology, writing – original draft, writing – review & editing, supervision, visualization, funding acquisition.

## Conflicts of interest

The authors declare no competing interests.

## Supplementary Material

SC-OLF-D4SC05115G-s001
