# Supplementary material for: HANNA: hard-constraint neural network for consistent activity coefficient prediction
Source: Chem Sci. 2024 Oct 31;15(47):19777–86. doi: 10.1039/d4sc05115g (PMC11575590; doi:10.1039/d4sc05115g)
Supplement: SC-015-D4SC05115G-s001 [file SC-015-D4SC05115G-s001.pdf]

Supplementary Information (SI) for Chemical Science.
This journal is © The Royal Society of Chemistry 2024

Supporting Information for HANNA: Hard-constraint Neural Network for Consistent Activity Coefficient Prediction

Thomas Specht,[†] Mayank Nagda,[‡] Sophie Fellenz,[‡] Stephan Mandt,[¶] Hans Hasse,[†] and Fabian Jirasek[*,†]

†*Laboratory of Engineering Thermodynamics (LTD), RPTU Kaiserslautern, Germany*

‡*Department of Computer Science, RPTU Kaiserslautern, Germany*

¶*Department of Computer Science, University of California, Irvine, CA, USA*

E-mail: *fabian.jirasek@rptu.de

Temperature distribution of data

Figure S.1 shows the distribution of the binary activity coefficients over temperature in the training set as well as in the validation and test set.

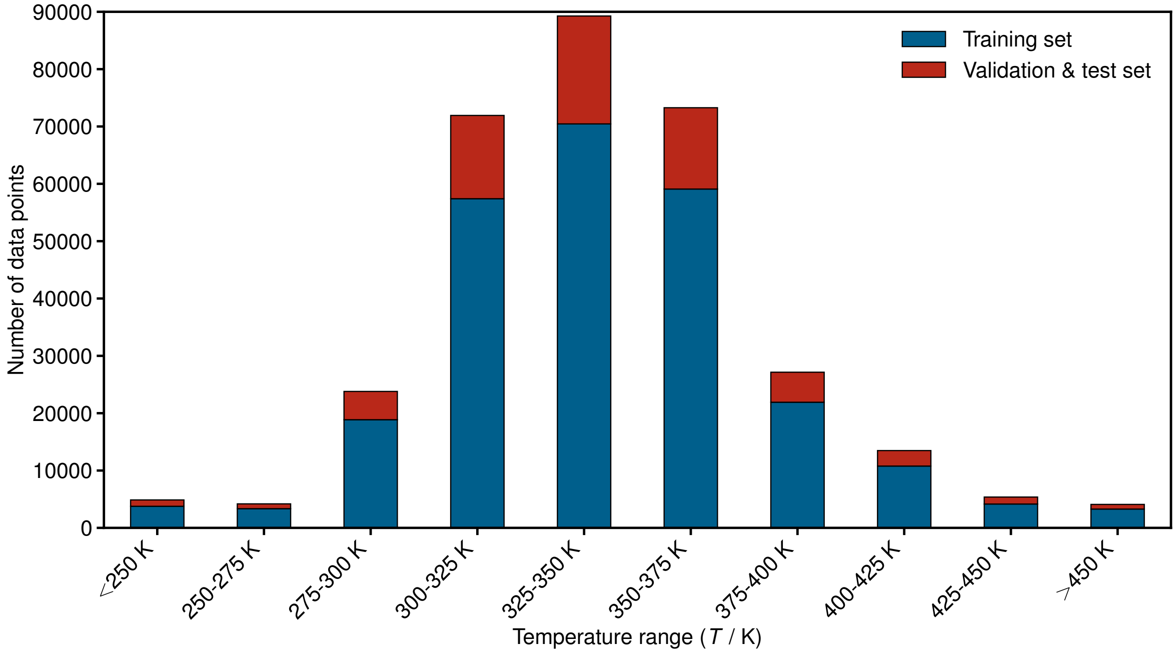

Figure S.1: Temperature distribution of the experimental data points for binary activity coefficients used in this work.

Figure S.2 shows the data point-wise MAE of the predicted logarithmic activity coefficients in boxplots on the UNIFAC horizon for HANNA and UNIFAC for different temperature intervals. HANNA shows more accurate results in all temperature intervals.

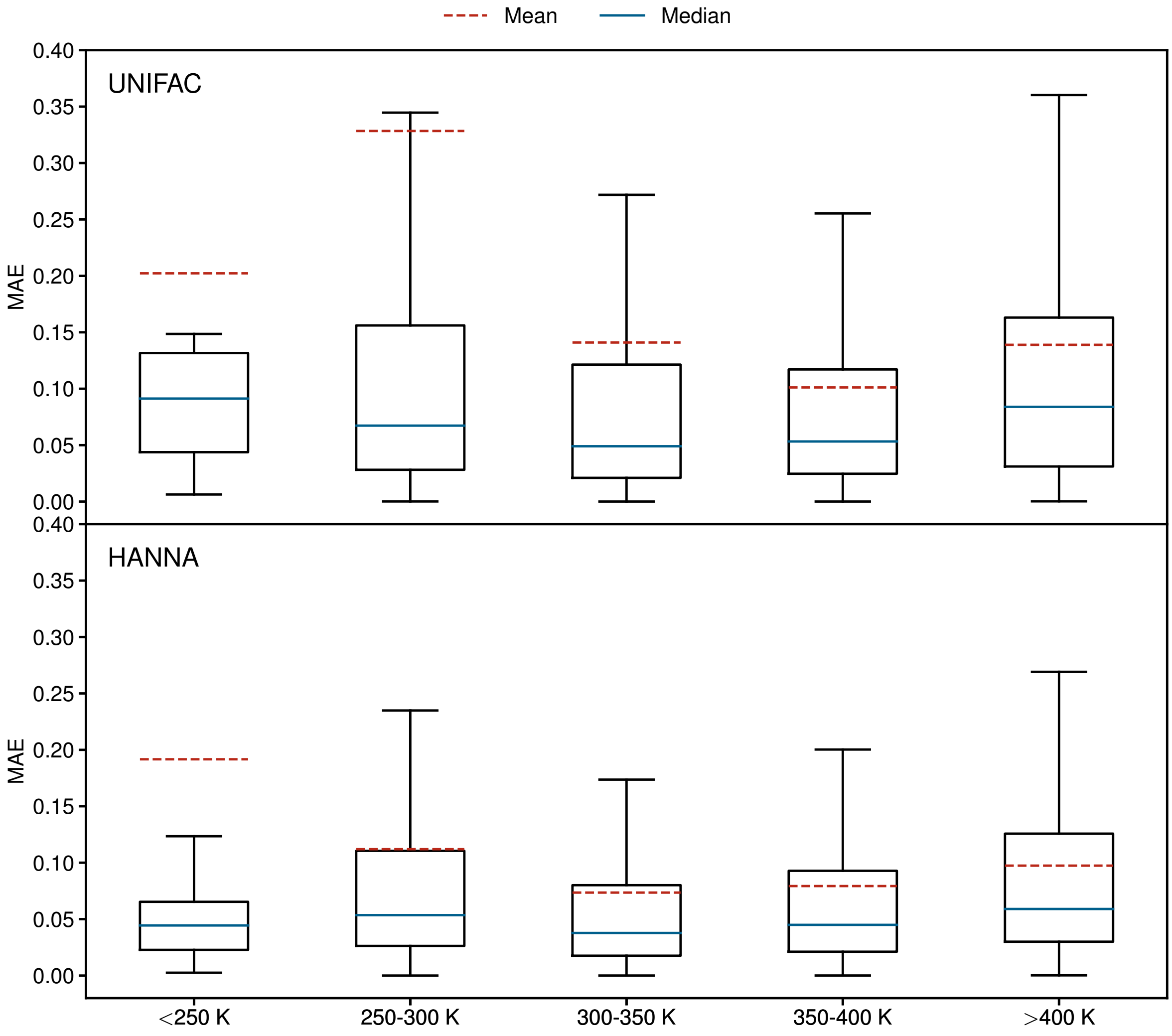

Figure S.2: Data point-wise MAE of the predicted logarithmic activity coefficients $\ln\gamma_i$ for HANNA and UNIFAC on the UNIFAC horizon for different temperature intervals on the test set.

Ablation studies

This section discusses the results of three ablation experiments to understand the importance of different parts of HANNA's architecture. Figure S.3 shows the architecture of the ablation models. For better comparison, if possible, the same number of nodes as in the final HANNA model was used, as well as the same number of layers. The number of nodes was only changed to accommodate the dimension due to a concatenation of features. In ablation model 1, the logarithmic activity coefficients $\ln\gamma_i$ are predicted directly without considering the thermodynamic constraints. We had to adapt the architecture slightly further to ensure an interaction modeling between the components. Therefore, the mixture embeddings $\boldsymbol{f}_\alpha(\boldsymbol{C}_1)$ and $\boldsymbol{f}_\alpha(\boldsymbol{C}_2)$ are concatenated to $\boldsymbol{C}_{\mathrm{mix},1} = [\boldsymbol{f}_\alpha(\boldsymbol{C}_1), \boldsymbol{f}_\alpha(\boldsymbol{C}_2)]$ and $\boldsymbol{C}_{\mathrm{mix},2} = [\boldsymbol{f}_\alpha(\boldsymbol{C}_2), \boldsymbol{f}_\alpha(\boldsymbol{C}_1)]$. Each of them is then processed through the property network f_ϕ to calculate $\ln\gamma_1$ and $\ln\gamma_2$, respectively.

In ablation model 2, the logarithmic activity coefficients $\ln\gamma_i$ are again modeled directly without the intermediate prediction of g^{E}. However, we now use the mixture embedding $\boldsymbol{f}_\alpha(\boldsymbol{C}_i)$ to predict $\ln\gamma_i$ in the property network f_ϕ. In ablation model 3, the embeddings $\boldsymbol{E}_i$ from ChemBERTa-2 are directly aggregated to $\boldsymbol{E}_{\mathrm{mix}}$ using the sum operation. The standardized temperature T^* as well as the product of both mole fraction $x_1(1-x_1)$ are concatenated to $\boldsymbol{E}_{\mathrm{mix}}$ to build $\boldsymbol{C}_{\mathrm{mix}}$, which is then processed by the "property prediction" network f_ϕ to calculate $g^{\mathrm{E}}_{\mathrm{NN}}$. g^{E} and the logarithmic activity coefficients are then derived in the same way as in HANNA, cf. Section *Development of HANNA* in the manuscript. Note that the activity coefficients are still modeled as equivariant properties in all ablation models due to our deep-set architecture.

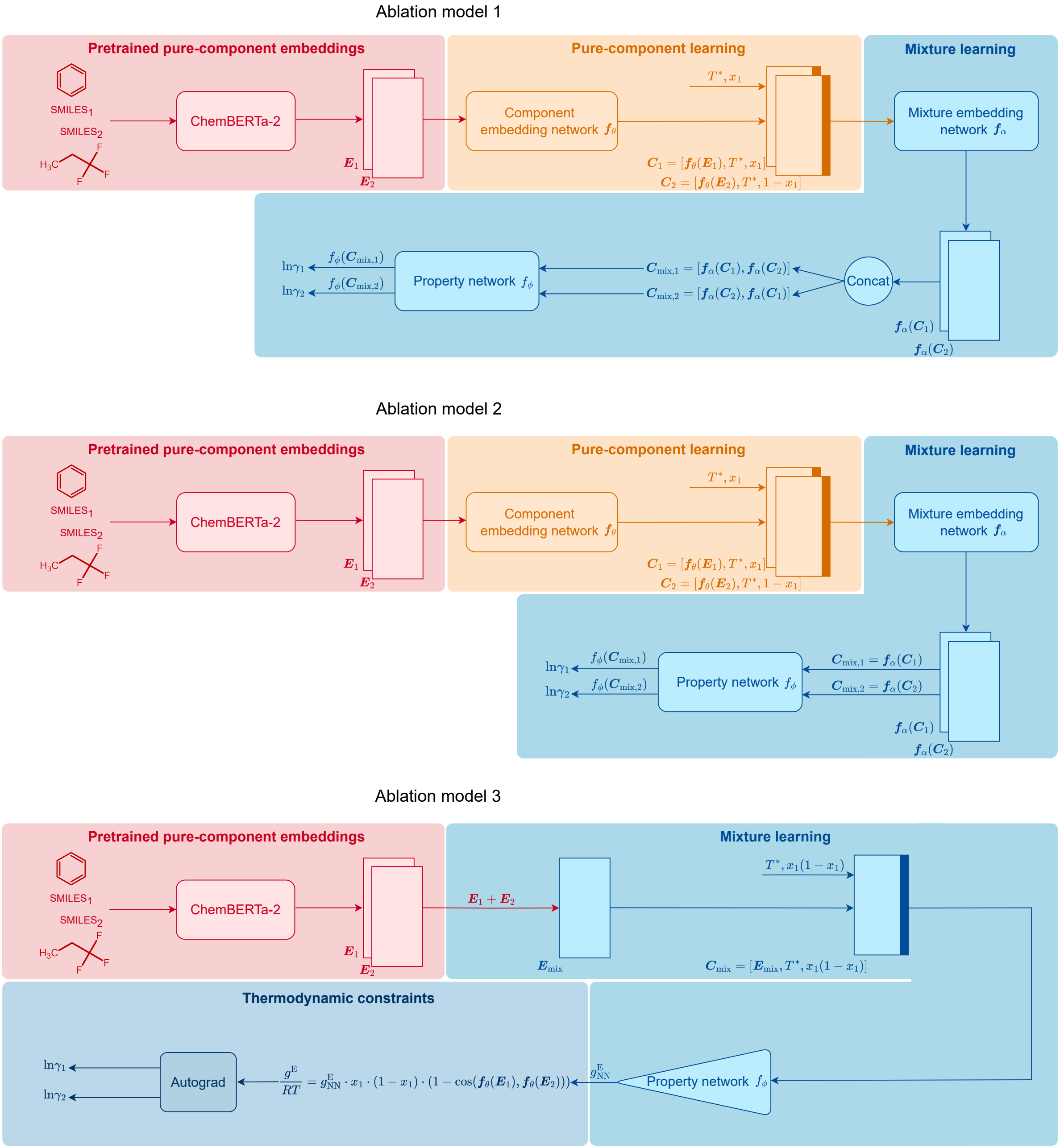

Figure S.3: Architectures of the three ablation models.

Figure S.4 (left) shows the training and validation SmoothL1Loss over the epochs for HANNA and the three ablation models. Ablation model 1 shows nearly the same loss as HANNA, whereas ablation model 2 shows a significant score deterioration. These results underpin that interaction modeling is necessary, either through a concatenation of the features of the components as in ablation model 1 or through a summation as in HANNA or ablation model 3. Furthermore, the results show that the model flexibility is not overly restricted by hard-constraining its predictions to the thermodynamically consistent solution space.

Figure S.4 (right) shows the mean squared deviation of the model predictions from the Gibbs-Duhem equation, cf. Equation (2) in the manuscript, for the training and validation set over the epochs for HANNA and the three ablation models. As expected, HANNA and ablation model 3 show zero error over all epochs since Gibbs-Duhem consistency is strictly enforced in their network architectures. In contrast, strict Gibbs-Duhem consistency is not obtained with the ablations models 1 and 2. Specifically, the loss of ablation model 1 decreases during the first epochs; the model obviously "learns" something about the Gibbs-Duhem equation during the training, but only until a certain threshold is reached. We can assume that this is, among others, caused by inconsistencies in the experimental training data.[1] Hence, learning Gibbs-Duhem consistency solely based on experimental data seems unfeasible. Ablation model 2 also learns something about Gibbs-Duhem consistency during the first epochs, but then it shows instabilities.

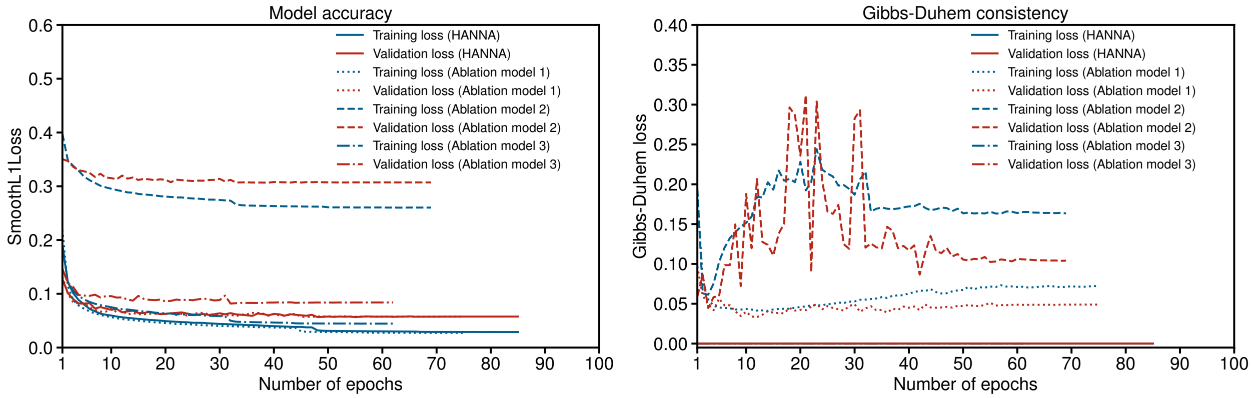

Figure S.4: Left: SmoothL1Loss for the training and validation set over the epochs for HANNA and the three ablation models. Right: Mean squared deviation of model predictions from the Gibbs-Duhem equation, cf. Equation (2) in the manuscript, for the training and validation set over the epochs for the four models. The end of the training is determined by the validation loss, cf. Section *Data splitting, training, and evaluation of the model* in the manuscript for details. HANNA and ablation model 3 show zero deviation from the Gibbs-Duhem equation throughout.

Figure S.5 shows the system-specific MAE of HANNA and the three ablation models on the test data. Overall, HANNA shows the best performance together with ablation model 1, which, however, does not give physically consistent predictions.

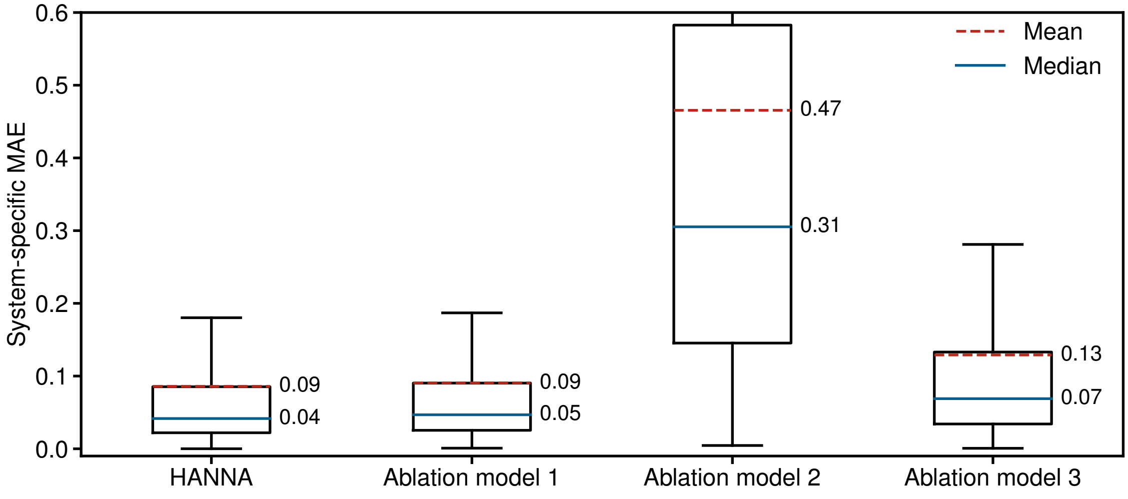

Figure S.5: System-specific MAE of the predicted logarithmic activity coefficients $\ln\gamma_i$ from the test set with HANNA and the three ablation models. The upper whisker of ablation model 2 ends at an system-specific MAE of 1.24.

Extrapolation to unknown components

Figure S.6 compares HANNA and UNIFAC for predicting the systems from the test set where one component is entirely unknown to HANNA. Furthermore, the results from HANNA for all systems with one unknown component from the complete test set (complete horizon) are shown. As discussed in the manuscript, there could, in principle, be another class of test systems in which both components are unknown to HANNA. However, since this was the case for precisely a single system within the UNIFAC horizon, the respective results are omitted here. Note that we can distinguish these cases only for HANNA since the training set of UNIFAC has not been disclosed.

The boxplots demonstrate that HANNA is significantly more reliable in extrapolating to systems containing unknown components than UNIFAC. For both models, the scores shown here are significantly worse than those shown in Figure 2 of the manuscript, which also covers the test data points where only the system was unknown to HANNA. This observation might be explained considering two facts: first, systems containing water are heavily over-

represented in the data sets shown in Figure S.6 (e.g., 70% of the systems of the UNIFAC horizon contain water), and systems with water are known to be rather tricky to describe. Second, data for activity coefficients at infinite dilution are heavily over-represented in the data sets shown in Figure S.6 (e.g., for 80% of the systems of the UNIFAC horizon, only data for activity coefficients at infinite dilution are available), which are, again, more difficult to predict than data at finite concentrations, among others, due to the high experimental uncertainty of activity coefficients at infinite dilution.

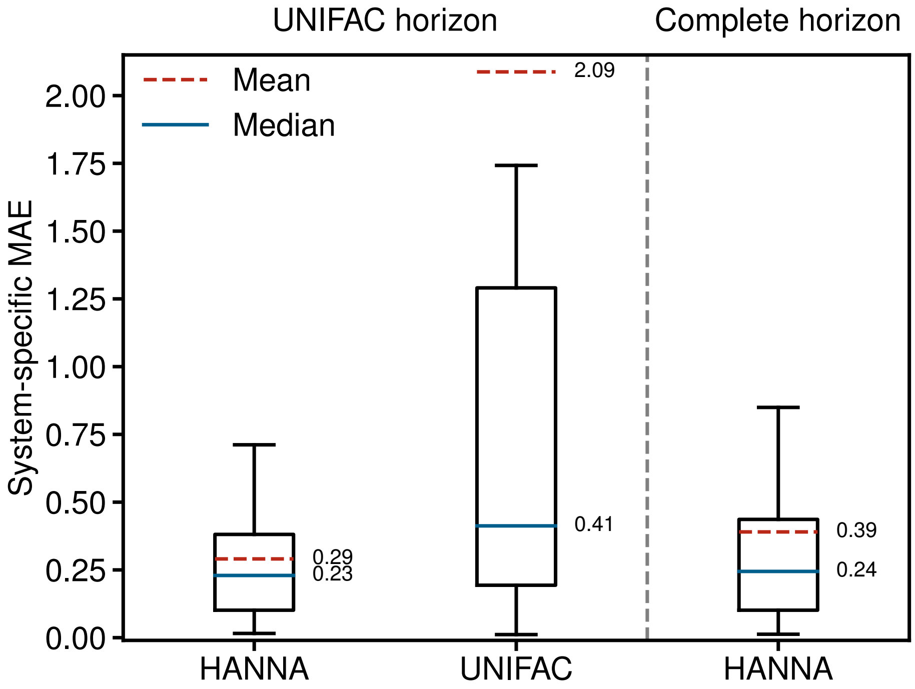

Figure S.6: System-specific MAE of the predicted logarithmic activity coefficients $\ln\gamma_i$ from HANNA and UNIFAC for the case that one component is unknown in the system. Left: results for the 40 out of 1658 systems from the test set that can also be predicted with UNIFAC (UNIFAC horizon). Right: results for the 56 out of 3502 systems from the complete test set (complete horizon).

Hyperparameter optimization

Table S.1 shows the varied hyperparameters in developing HANNA and the SmoothL1Loss achieved on the validation data. Model 7 was used throughout the manuscript.

Table S.1: Varied hyperparameters in the development of HANNA and their influence on the validation loss. λ is the weight decay in the ADAMW optimizer.

Model No.	λ	Number of nodes	Initial learning rate	SmoothL1Loss
1	0.01	128	0.001	0.0574
2	0.01	128	0.0005	0.0639
3	0.001	128	0.001	0.0576
4	0.001	128	0.0005	0.0641
5	0.0001	128	0.001	0.0576
6	0.0001	128	0.0005	0.0641
7	0.01	96	0.001	0.0567
8	0.01	96	0.0005	0.0579
9	0.001	96	0.001	0.0579
10	0.001	96	0.0005	0.0573
11	0.0001	96	0.001	0.0569
12	0.0001	96	0.0005	0.0570
13	0.01	64	0.001	0.0615
14	0.01	64	0.0005	0.0710
15	0.001	64	0.001	0.0584
16	0.001	64	0.0005	0.0705
17	0.0001	64	0.001	0.0588
18	0.0001	64	0.0005	0.0705

Results for different seeds

This section compares results for different splittings of the systems from the complete dataset into training, validation, and test sets. Figure S.7 shows boxplots for the system-specific MAE on the different test sets, indicated by different specified seeds, on the UNIFAC horizon. High robustness of HANNA is observed.

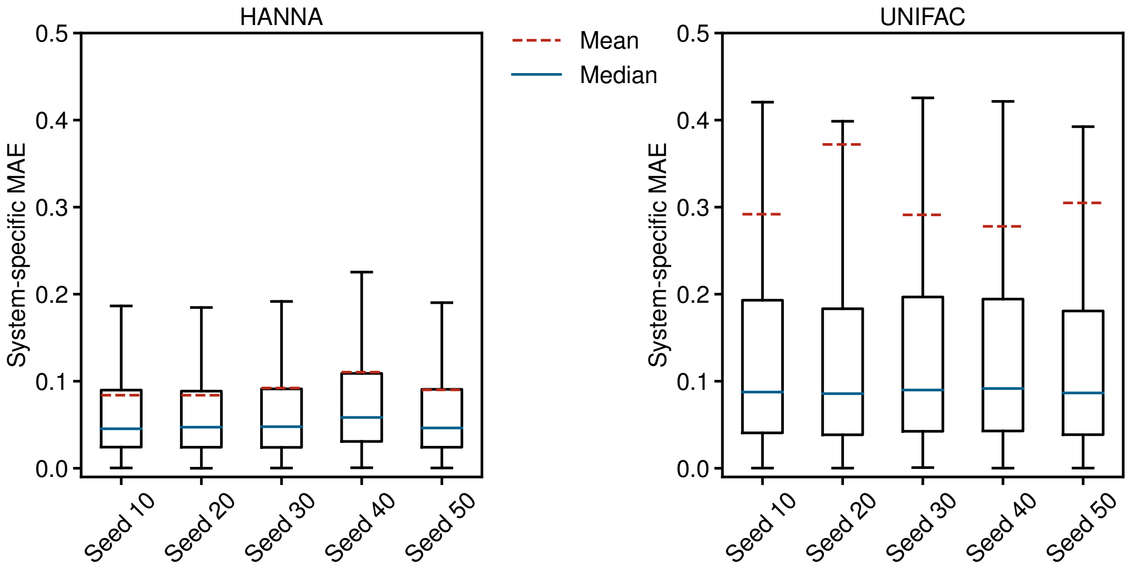

Figure S.7: System-specific MAE of HANNA (left) and UNIFAC (right) on the UNIFAC horizon for different test sets, defined by specifying different seeds in the data split. Seed 10 was used throughout the manuscript.

Improved tokenization of ChemBERTa-2

The first step of ChemBERTa-2 is a tokenization of the SMILES used as input. For example, the SMILES "CCO" representing ethanol is split into "C", "C", and "O". However in our study, we found that the tokenization of some SMILES were incorrect, e.g., "CCCl" is incorrectly split into "C", "C", "C", i.e., wrongly substituting the "Cl" by a "C". We have therefore used our own tokenizer to correctly split the SMILES. Our tokenizer processes SMILES strings by splitting them into distinct tokens, specifically capturing atom representations enclosed in square brackets (e.g., [Xe]) and two-letter elements like "Br" and "Cl". The detailed code is provided in our Github.[2]

References

(1) Gmehling, J. et al. *Chemical thermodynamics for process simulation*, 2nd ed.; John Wiley & Sons, 2019.

(2) HANNA Github. https://github.com/tspecht93/HANNA, Last acessed: 23.09.2024.
